# Supplementary figures and images for: Observations of genetic differentiation between the fall armyworm host strains
Source: PLoS One. 2022 Nov 11;17(11):e0277510. doi: 10.1371/journal.pone.0277510 (PMC9651577; doi:10.1371/journal.pone.0277510)

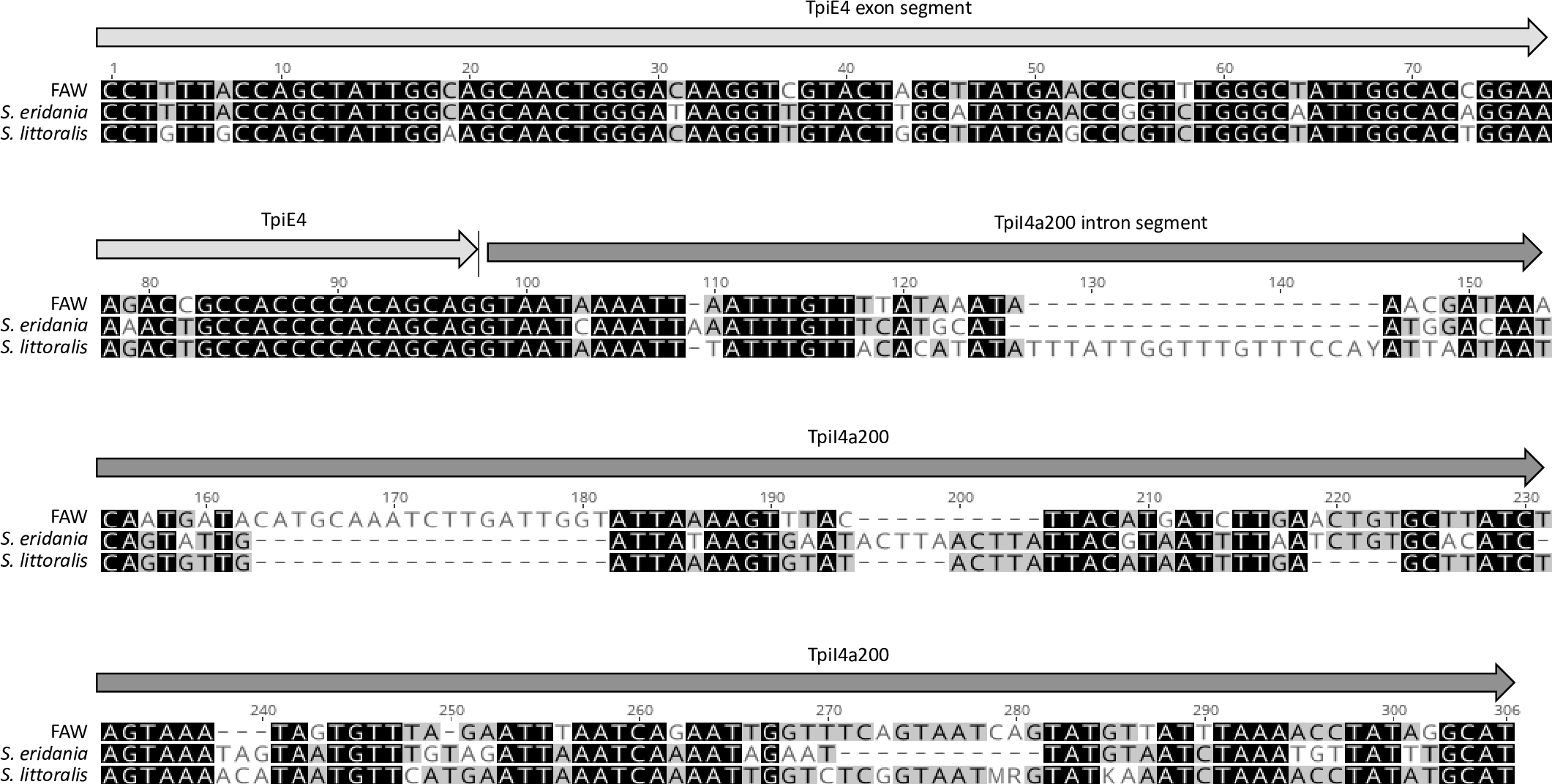

Supplement: S1 Fig — (TIF) [file pone.0277510.s001.tif]

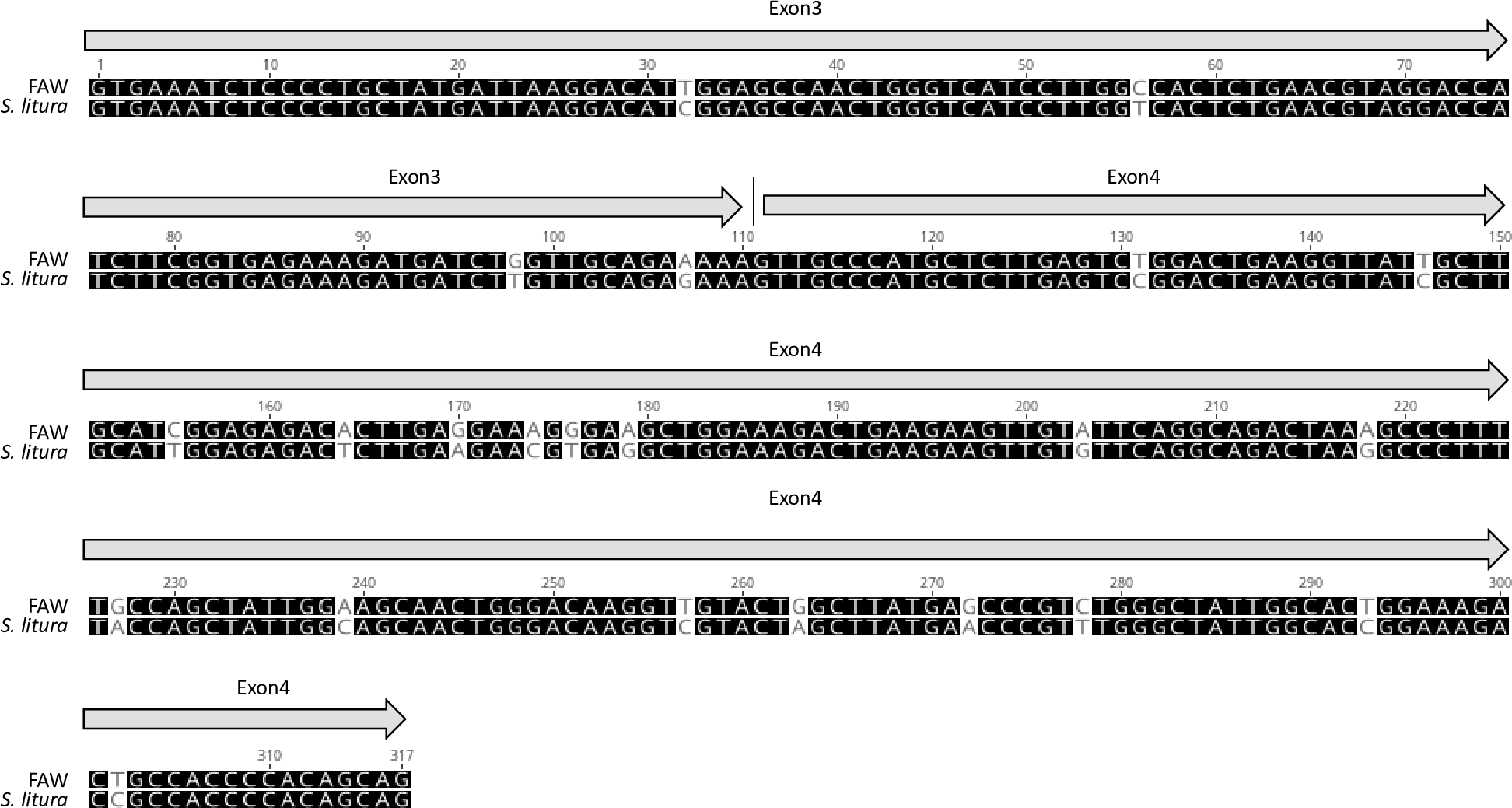

Supplement: S2 Fig — The S. litura sequence was obtained from Genbank accession number XM022977018. (TIF) [file pone.0277510.s002.tif]
