# Supplementary material for: Observations of genetic differentiation between the fall armyworm host strains
Source: PLoS One. 2022 Nov 11;17(11):e0277510. doi: 10.1371/journal.pone.0277510 (PMC9651577; doi:10.1371/journal.pone.0277510)
Supplement: S1 Table — ClanC or CHost are designated C while ClanR or RHost are designated R. (DOCX) [file pone.0277510.s003.docx]

Supplementary Table S1. Calculations of *F_ST_* for populations categorized by host use (C_Host_, R_Host_) or phylogenetic groups (ClanC, ClanR). ClanC or C_Host_ are designated C while ClanR or R_Host_ are designated R.

| POPULATION 1 | POPULATION 2 | *F_ST_* (phylogenetic groups) | *F_ST_* (plant host) |
| --- | --- | --- | --- |
| Argentina C | Brazil C | 0.047 | 0.046 |
| Argentina C | Florida Larva C | -0.027 | 0.014 |
| Argentina C | Florida Traps C | 0.071 | 0.119 |
| Brazil C | Florida Larva C | 0.046 | 0.099 |
| Brazil C | Florida Traps C | 0.080 | 0.108 |
| Florida Traps C | Florida Larva C | 0.102 | 0.208 |
|  | **C vs C mean** | **0.053** | **0.099** |
|  |  |  |  |
| Argentina R | Brazil R | 0.002 | -0.005 |
| Argentina R | Florida Larva R | 0.001 | 0.084 |
| Argentina R | Florida Traps R | 0.002 | -0.002 |
| Brazil R | Florida Larva R | 0.053 | 0.164 |
| Brazil R | Florida Traps R | 0.012 | 0.050 |
| Florida Traps R | Florida Larva R | 0.004 | 0.054 |
|  | **R vs R mean** | **0.012** | **0.057** |
|  |  |  |  |
| Argentina C | Argentina R | 0.483 | 0.272 |
| Argentina C | Brazil R | 0.406 | 0.189 |
| Argentina C | Florida Larva R | 0.566 | 0.466 |
| Argentina C | Florida Traps R | 0.512 | 0.334 |
| Brazil C | Argentina R | 0.352 | 0.336 |
| Brazil C | Brazil R | 0.291 | 0.261 |
| Brazil C | Florida Larva R | 0.422 | 0.494 |
| Brazil C | Florida Traps R | 0.381 | 0.387 |
| Florida Larva C | Argentina R | 0.479 | 0.140 |
| Florida Traps C | Argentina R | 0.505 | 0.490 |
| Florida Larva C | Brazil R | 0.401 | 0.072 |
| Florida Traps C | Brazil R | 0.429 | 0.416 |
| Florida Larva C | Florida Larva R | 0.562 | 0.341 |
| Florida Larva C | Florida Traps R | 0.508 | 0.206 |
| Florida Traps C | Florida Larva R | 0.586 | 0.658 |
| Florida Traps C | Florida Traps R | 0.532 | 0.522 |
|  | **C vs R mean** | **0.463** | **0.349** |
