# Supplementary material for: Observations of genetic differentiation between the fall armyworm host strains
Source: PLoS One. 2022 Nov 11;17(11):e0277510. doi: 10.1371/journal.pone.0277510 (PMC9651577; doi:10.1371/journal.pone.0277510)
Supplement: Attachment — Submitted filename: reviewer rebuttal.docx [file pone.0277510.s005.docx]

Reviewer #2

<<<Line 270-273: I encourage the author to move this sentence down to around line 285. Currently, the authors talk about clan-host comparisons, then begin presenting findings about geographic differences, then go back to clan-host comparisons. This is not easy for the reader to follow. Consider revising this section to make these two ideas distinct.>>>

The changes were made as suggested with lines 270-281 rearranged and modified. In addition an error was found in the numbering of the figures and this was corrected.

<<<Line 347: I agree that these two strains are experiencing differential selection pressures, but since this is correlative and not causative, it can not necessarily be linked to differential host use.>>>

Changed the necessary linkage to a speculative one by replacing “because” with “that could be related to” (line 345).

<<<Supplementary Table S1: In the bottom of the table where C vs R comparisons are being made, the author should consider reordering the comparisons so that population 1 is all individuals of the C- strain and population 2 is the R-strain comparison. The data will be the same, but this would be a bit easier for the reader to interpret.>>>

Done

Reviewer #3: 

General criticism
<<<The abstract and introduction discussed the FAW as a new pest of the eastern hemisphere and that only one stain was found compared to the Americas. Does this study have any implications for to FAW in the eastern hemisphere? If not, it seems an unnecessary given the FAW is still a major pest in the Americas where this research was conducted.>>>

Whether the strains exist and, if so, which are present in the Eastern Hemisphere has important ramifications to assessing crops at risk. Since this paper provides evidence supporting the legitimacy of the strain concept, I believe it is relevant to mention the situation in the Eastern Hemisphere. This rationale is noted in lines 33-35 and 37-39.

<<<How is this research different from other studies using the tpi gene? Lines 37-40 indicates other studies have used the tpi gene to differentiate between FAW strains.>>>

Past studies provided the empirical support for the use of Tpi SNPs as strain markers. The current study describes the phylogenetic relationships associated with the strains as defined by Tpi. This rationale is described in lines 81-92.

<<<How does this specific research further or alter the debate about the validity of calling these separate host stains? The study clearly advocates for the existence of different host strains, so is this study the end of the debate?>>>

This study provides genetic evidence supporting the existence of the strains and provides a phylogenetic description of how the strains differ. Whether it ends the debate on strains is for the scientific community to decide.

Specific criticism
<<<Line 61: has should be have>>>

Done (line 62)

<<<Line 343: Reference needs to be numerical>>>

Done (line 320)

<<<Line 408: plant hosts should be host plants>>>

Done (line 412)

<<<Lines 418-420 say host use was only difference between host strains from Americas. Does this this contradict Lines 70-72 which mention differences in pheromones, reproduction, and mating between strains?>>>

No. Strain differences in pheromones, reproduction, and mating have been described, but only for specimens derived from North American populations. Lines 418-420 (now 389-390) states that “differential host use is the only trait demonstrated in host strain populations from both American continents”.
